# Fornix degeneration in risk factors of Alzheimer's disease, possible trigger of cognitive decline

**DOI:** 10.1016/j.cccb.2023.100158

**Published:** 2023-01-17

**Authors:** María Lacalle-Aurioles, Yasser Iturria-Medina

**Affiliations:** a Department of Neurology and Neurosurgery, Montreal Neurological Institute, McGill University, Montréal, QC H3A 2B4, Canada; b Ludmer Centre for Neuroinformatics and Mental Health, McGill University, Montreal, Canada; c McConnell Brain Imaging Centre, McGill University, Montreal, Canada

**Keywords:** White matter, Neuroinflammation, Cerebrovascular pathology, Blood-brain barrier, Diffusion tensor imaging, Galectin-3, CVRFs, cardiovascular risk factors, DTI, difussion tensor imaging, NODDI, neurite orientation dispersion and density imaging, TBI, traumatic brain injury, WM, white matter

## Abstract

Risk factors of late-onset Alzheimer's disease (AD) such as aging, type 2 diabetes, obesity, heart failure, and traumatic brain injury can facilitate the appearance of cognitive decline and dementia by triggering cerebrovascular pathology and neuroinflammation.

White matter (WM) microstructure and function are especially vulnerable to these conditions. Microstructural WM changes, assessed with diffusion weighted magnetic resonance imaging, can already be detected at preclinical stages of AD, and in the presence of the aforementioned risk factors. Particularly, the limbic system and cortico-cortical association WM tracts, which myelinate late during brain development, degenerate at the earliest stages. The fornix, a C-shaped WM tract that originates from the hippocampus, is one of the limbic tracts that shows early microstructural changes. Fornix integrity is necessary for ensuring an intact executive function and memory performance. Thus, a better understanding of the mechanisms that cause fornix degeneration is critical in the development of therapeutic strategies aiming to prevent cognitive decline in populations at risk. In this literature review, i) we deepen the idea that partial loss of forniceal integrity is an early event in AD, ii) we describe the role that common risk factors of AD can play in the degeneration of the fornix, and iii) we discuss some potential cellular and physiological mechanisms of WM degeneration in the scenario of cerebrovascular disease and inflammation.

## Highlights

• Alzheimer's disease has a multifactorial etiology.
• Cerebrovascular dysfunction and white matter inflammation are early factor in AD.
• The fornix is highly vulnerable to vascular deficits and inflammation.
• Fornix degeneration occurs in AD but also in risk factors of AD.
• Fornix degeneration can facilitate dementia in populations at risk.

## 1 Introduction

Late-onset Alzheimer's disease (AD) is the most common neurological disorder in aged populations and it is expanding rapidly due to an increased lifespan and, presumably, to changes in our life style [1,2]. Presence of cardiovascular risk factors (CVRFs) in midlife, the absence of physical exercise in our daily routine, or traumatic brain injuries (TBI) may facilitate the development of AD in the elderly, in part by triggering cerebrovascular disease and neuroinflammation [3,4].

White matter (WM) is known to be especially susceptible to vascular pathology, supposedly, due to a lower microvascular density and reduced blood supply compared to the gray matter [5], [6], [7]. Interestingly, in recent years, a growing body of literature proposes microstructural WM changes as one of the earliest events in late-onset AD [8,9]. Microstructural WM changes in AD patients are predominantly found in parietotemporal regions (cingulate and parahippocampal WM tracts), the limbic system (uncinate fasciculus and fornix), and some parts of the corpus callosum, especially in posterior regions such as the splenium [10], [11], [12], [13], [14], [15], [16]. Among these fibers, the limbic system and the cortico-cortical association tracts seem to be more vulnerable [17], [18], [19].

The fornix is the main inflow/outflow information pathway of the hippocampus, making it a key structure in learning and memory processes. Reduced forniceal integrity already appears at preclinical phases of AD (reviewed in Nowrangi and Rosenberg [20]) and under risk factors of AD: aging [21,22], obesity [23], [24], [25], type 2 diabetes [26], [27], [28], heart failure [29] or TBI [30,31]. However, it is still unknown whether fornix degeneration may facilitate cognitive decline in populations at risk of developing AD. Moreover, the cellular and physiological mechanisms involved in fornix degeneration remain elusive.

We performed a search in PubMed® database to select original research articles, case reports, and review papers reporting microstructural WM changes in the fornix among AD patients or subjects with CVRFs or TBI. Keywords used in the search included: “white matter”, “fornix”, “diffusion tensor imaging”, “type 2 diabetes”, “obesity”, “heart failure”, and “traumatic brain injury”. Additionally, a search for WM degeneration mechanisms included: “blood-brain barrier”, “white matter inflammation” and “Galectin-3″.

In this literature review, we deepen the idea of WM damage of the fornix as an early event in AD, and summarize the literature reporting forniceal damage in risk factors of AD. Finally, we describe key vascular and inflammatory mechanisms that can potentially trigger forniceal WM alterations and facilitate cognitive deficits.

## 2 Cerebrovascular pathology and white matter changes in Alzheimer's disease

AD has been traditionally considered a neurodegenerative disorder with amyloid-β (Aβ) plaques and neurofibrillary tangles (composed of hyperphosphorylated tau) as primary histological hallmarks. Since the early 1990s, the amyloid hypothesis has motivated the vast majority of clinical trials in AD [32], even though in 1984 it was accepted by the scientific community that a relevant vascular component contributes to AD [33].

Recently, the vascular component of AD has regained prominence. Epidemiological studies have associated some CVRFs (i.e., smoking, diabetes and obesity) with a higher incidence of AD and, presumably, clinical strategies focused on CVRF prevention could reduce the predicted prevalence of AD in the long term [34,35].

A compromised cerebrovascular function is now recognized as a critical event in the development of late-onset AD, with up to 80% of AD patients showing vascular pathology [5,[36], [37], [38], [39], [40], [41], [42]]. Moreover, causal multifactorial studies point to the vascular component as a disease triggering factor [43,44]. According to these studies, late-onset AD would be caused by the complex interplay among multifactorial interactions, not by a unique dominant biological factor (e.g., vascular or Aβ), and most-likely, the brain vasculature would be the initial pathologic event. Yet, the specific interplay between vascular and neuronal pathologies in AD is poorly understood.

In addition, neuroimaging studies of diffusion tensor imaging (DTI) have pointed to disrupted functional networks, due to microstructural WM changes, as a possible cause of the earliest cognitive impairments in AD patients.

In the WM, DTI studies rely on the highly ordered (anisotropic) water diffusion that results from the unique geometric organization of axonal fiber bundles. Changes in DTI parameters (e.g., axial diffusivity (AxD), radial diffusivity (RD), mean diffusivity (MD) and fractional anisotropy (FA)) refer to the altered anisotropy and/or diffusivity of water molecules caused by changes in the WM microstructure [45,46]. MD and FA are the most used parameters in AD studies. MD corresponds to the average water diffusion within the region of interest independent of the tissue direction. FA is the main parameter of anisotropic (directional) diffusion, with higher values reflecting increased directionality of diffusion, and mostly reflects the alignment of the cellular structures and the degree of axonal fiber bundle organization [47], [48], [49]. Several factors such as reduced fiber density, decreased myelination in fiber tracts, gliosis, inflammation, and ischemic processes can modulate the barriers to free water diffusion, reduce anisotropy and/or increase water diffusivity in the WM [50,51]. Although the specific causes for DTI parameter changes remain unclear, evidence that those changes occur early in AD supports the brain disconnection hypothesis in these patients [9,16,[52], [53], [54], [55], [56]]. According to this hypothesis, the partial loss of WM fiber integrity would contribute to the development of dementia by interfering with a proper signaling between interconnected brain regions [57], [58], [59]. A complex task such as memory retrieval requires the coordinated activity of functionally connected regions, thus, the partial disconnection of these regions could lead to a loss of function [60].

Interestingly, AD patients with a strong vascular component show more severe microstructural WM changes than those with a subtle vascular component [61], which suggest that WM integrity is highly susceptible to cerebrovascular deficits. Changes in water diffusivity at pre-symptomatic stages of AD can be assessed with DTI long before macrostructural WM changes, in the form of WM hyperintensities, can be detected with conventional magnetic resonance imaging (MRI), and before the presence of Aβ plaques and neurofibrillary tangles (reviewed in Sachdev et al. [58]). Moreover, more advanced and sensitive imaging techniques able to provide information about neurite microstructure (neurite orientation dispersion and density imaging (NODDI)) have detected microstructural WM changes in groups of preclinical AD patients in which DTI failed to find WM deficits [62].

The disconnection hypothesis has added one more level of complexity to the understanding of AD etiology but, on the other hand, comprehending the physiological mechanisms that trigger WM changes and the link with vascular pathology may shed light on the relationship between the cerebrovascular dysfunction seen in many risk factors of AD and the neurodegeneration finally leading to AD.

A better understanding of these mechanisms is also essential for novel therapies aiming to rescue dysfunctional brain circuits to succeed. Namely, deep brain stimulation of the fornix (DBS-f). DBS is a neurosurgical procedure able to modulate the activity of brain circuits through electrical stimulation delivered by electrodes that are implanted in target areas in the brain [63]. Neuromodulation of the fornix by DBS is currently under research for memory rescue in patients with AD [64]. However, even though initial stages of the clinical trial showed a certain delay in the progression of cognitive decline in AD patients, later results from more advanced stages of the trial have failed to confirm such benefits as a group effect and suggest benefits in only a subgroup of AD patients (i.e., ≥ 65 years) [65,66]. According to the authors, the inhomogeneity in functional and structural deficits between subjects could have been among the limiting factors of the study. For this reason, it is important to be able to efficiently identify the subpopulations of AD patients that can potentially benefit from this type of procedure.

## 3 Fornix anatomy and its role in learning and memory processes

**Fig. 1:**
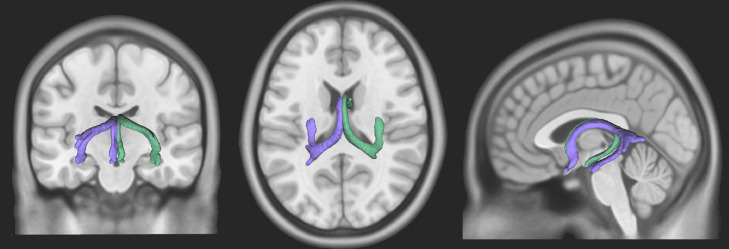
Three-dimensional representation of the fornix of a healthy adult subject. Coronal (left), axial (center) and sagittal (right) views, overlapped on a T1-weighted magnetic resonance image. DSI studio software was used for visualization (dsi-studio.labsolver.org).

The fornix is an arch-shaped WM structure located under the corpus callosum and above the thalamus (Fig. 1). It is composed of two symmetric bands (one per hemisphere) that are divided into three anteroposterior anatomical segments: columns, body and crura. The fornix connects the hippocampus with the diencephalon and the basal forebrain, and interconnects bilateral hippocampi across the hippocampal commissure [67]. In an anterograde pathway (septo-hippocampal pathway), the fornix carries the efferent axons of pyramidal cells in the subiculum and cornu ammonis 1 (CA1) and 3 (CA3) of the hippocampus. In a retrograde pathway, the fornix carries the afferent cholinergic, GABAergic and glutamatergic projections from the medial septum-diagonal band of Broca to the hippocampus, modulating the activity of neurons and interneurons [18,68,69].

The interruption of this circuitry as a consequence of fornix degeneration in aging or pathological conditions is believed to impair executive function and episodic memory consolidation [70], [71], [72], [73], [74]. Most evidence for fornix degeneration triggering learning and memory deficits comes from experimental animal models or case studies reporting forniceal lesions caused by infarction, traumatic injuries, or tumors (reviewed in Senova et al., [69]). These reports indicate that different aspects of the fornix are involved in distinct memory functions [75], [76], [77]. Namely, verbal and visuospatial memory information are thought to be primarily carried by the left and the right fornix, respectively. Additionally, while emotional and motivational learning would be mainly processed by lateral regions of the fornix, the medial aspect of this structure would be responsible of integrating object recognition within a spatial context. Bilateral lesions of the anterior columns of the fornix can lead to anterograde and retrograde amnesia.

The specific mechanism by which fornix degeneration impairs cognition and memory in humans are poorly understood, especially because, in most cases, lesions of the fornix are accompanied with lesions in other brain regions that could also be involved in learning and memory (i.e., the medial temporal lobe or the mammillary bodies). But experimental animal models suggest dysregulation of the cholinergic and GABAergic signaling as possible factors [78], [79], [80]. These neurotransmitters are key as they modulate cortical and hippocampal neuronal activity.

The role of the fornix in memory recall has also been studied in healthy volunteers by using DTI [45]. In a study with healthy young adults, higher FA in the fornix correlated with better recollection in scene and object recognition tasks [81]. In another study with healthy subjects, higher FA in the fornix correlated with better performance in the Doors and People Test, which assesses verbal and visual recall and recognition [82].

## 4 Changes in fornix microstructure, an early event in AD

WM degeneration in AD has been explained by two different mechanisms: Wallerian degeneration and the retrogenesis model [17,83,84]. The hypothesis based on Wallerian degeneration assumes secondary axonal damage following cortical neurodegeneration, but considering that the literature has failed to find a direct association between fornix degeneration and hippocampal atrophy [16,[85], [86], [87]], Wallerian degeneration would not be enough to explain early changes in the fornix.

On the contrary, the retrogenesis model assumes primary WM changes through myelin breakdown and axonal damage that develops in a reverse pattern of myelinogenesis during brain development [17,83,84]. This process would first affect late-myelinating fibers during brain development (limbic system and the cortico-cortical association tracts) due to their thinner myelin sheaths and, more severely, those of small dimeter such as the fornix [17], [18], [19].

The fact that fornix degeneration appears at preclinical phases of AD disease, and that it has a strategic location within the memory circuitry, makes it a good candidate for an early AD biomarker [20]. Several groups have found that amnesic mild cognitive impairment (aMCI) patients that later progressed to AD have reduced FA and increased RD in the fornix, compared with healthy controls [16,88,89]. Moreover, in a longitudinal study by Nowrangi et al., an increasing MD in the fornix was observed in aMCI patients over time, proposing this parameter as a good indicator of clinical progression [90]. Notably, forniceal WM changes have also been reported as a biomarker of conversion from cognitively normal subjects to aMCI [85]. In a cross-sectional study, Zhuang et al. reported that early aMCI subjects (cognitively normal subjects that progressed to aMCI within the 2 years of the study) already showed impaired WM in the fornix when they were cognitively normal. Those microstructural changes were extended to the parahippocampal WM tract, cingulum and uncinate fasciculus in late aMCI patients. Oishi et al. and Fletcher et al. have also reported that WM changes in the fornix predict conversion from cognitively normal subjects to aMCI [72,91].

Whether microstructural WM changes in the fornix are the cause or consequence of the neurodegeneration occurring in the hippocampus, the main structural biomarker of neurodegeneration proposed by the National Institute on Aging-Alzheimer's Association [92], has been largely debated. However, forniceal changes have been detected in early aMCI patients in absence of hippocampal atrophy [85], which suggest that WM alterations in this tract are independent from neuronal damage in the hippocampus and may occur directly within the axons, supposedly, by some myelin pathology or WM inflammation [93,94]. In line with this hypothesis, a study performed in cognitively normal elderly subjects with hippocampal atrophy and decreased FA in the fornix reported that changes in fornix microstructure preceded hippocampal atrophy when a sequential relationship was evaluated [86]. Accordingly, other studies have also failed to find an association between WM changes in the fornix and atrophy of the medial temporal lobe (entorhinal cortex or hippocampus) in aMCI or early AD patients [16,87]. However, there is, indeed, an association between fornix degeneration and hippocampal atrophy when looking at the specific hippocampal area harboring the cell bodies of forniceal axons. In two studies by Wiss et al. and Lee et al., performed in aMCI and early AD patients, decreased FA in the fornix was associated exclusively with decreased volume in the subiculum and CA1, but not with other hippocampal subfields [70,95]. The highly restricted location of gray matter atrophy associated with fornix degeneration suggests that neuronal death in the subiculum or CA1 could be due to delayed apoptosis or retrograde degeneration similar to that described in axotomy [96], [97], [98].

The association between WM changes in the fornix and the main hallmark of AD (Aβ deposition in the brain) has been explored by some authors. Reduced FA in the fornix was correlated with Aβ deposition when assessed with positron emission tomography in both cognitively normal and MCI subjects [99,100]. Also, in a cognitively normal population, lower Aβ42/p-tau181 ratios in the cerebrospinal fluid, a highly specific biomarker of AD [101], corresponded with altered WM integrity in the fornix, which also was associated to a lower performance in cognitive tests [102]. In a group of healthy subjects carrying mutations for presenilin and apolipoprotein (at high risk of developing a familial form of AD), fornix integrity was already compromised in absence of hippocampal atrophy, suggesting that forniceal changes may precede and be independent of hippocampal atrophy also in the familial form of AD [103]. However, the physiological mechanisms underlying forniceal damage in these subjects may not be comparable to the ones in late-onset AD due to the unidentified effects that these mutations may have in fornix structure during brain development.

APOE ε4 allele carriers, the strongest genetic factor known to increase the risk of late-onset AD [104,105], have also shown microstructural WM changes in the fornix at pre-symptomatic phases of the disease [59,94]. In this case, the aberran deposition of cholesterol in oligodendrocytes and the reduced axonal myelination, suposedly caused by cholesterol homeostasis and transport dysregulation, could be a major factor of WM degeneration and loss of function [106].

All these studies support that WM changes in the fornix are present at a very early stage of the disease, and often precede the structural alterations occurring in the hippocampus, but it remains unclear what causes forniceal degeneration at such early stages. Chronic hypoperfusion, ischemic processes, neuroinflammation, demyelination and axonal loss are some putative factors proposed by DTI studies that can alter the microstructure of the WM tracts in AD [49,107,108]. Actually, WM degeneration (independent of gray matter lesions) has been confirmed by post-mortem studies [109], [110], [111]. Demyelination, astrogliosis, axonal and oligodendrocyte loss in the deep WM have been reported from the autopsies.

## 5 Risk factors of AD can affect fornix microstructure through cerebrovascular pathology and neuroinflammation

Brain network dynamics are modulated by daily life factors such as education, diet, and physical exercise [112]. Over time, all of these factors will also mediate the degenerative process. Several risk factors of late-onset AD are known to impair the WM microstructure by triggering cerebrovascular pathology and neuroinflammation. Here, we describe some of the risk factors of AD in which the fornix has been reportedly affected. The fornix could be especially vulnerable to these pathological events not only because it is a part of the late-myelinating fibers of small diameter [17], but also because of its marked metabolic demand in comparison with other WM fibers [113].

### 5.1 Aging

Aging is the primary risk factor of neurodegeneration and dementia. Most DTI-based studies agree on WM microstructure being altered in elderly people [21,22]. The vast majority of studies performed with DTI have reported changes in integrity and density in the fornix (reviewed by Douet and Chang, [74]). Those changes are thought to be a consequence of local fiber degeneration and less likely a secondary event following hippocampal degeneration [114]. Indeed, recent studies using NODDI and quantitative magnetization transfer (qMT, an image modality sensitive to microglia-mediated inflammation) have reported that aging was associated with apparent glia but not neurite density damage in the fornix and have also confirmed that fornix WM glia damage causes hippocampal gray matter damage during age-dependent limbic decline [115].

Impaired cerebrovascular hemodynamics and inflammation are some of the age-related factors that can contribute to WM damage. The increased pulse pressure waves penetrating the cerebral microcirculation as a result of reduced arterial myogenic responses, may contribute to WM damage, and it has been reported to precipitate cognitive deficits in the elderly [116], [117], [118]. Also, the so-called ‘inflammaging’, potentially caused by an age-triggered imbalance in cytokine homeostasis, can precipitate WM degeneration [119].

### 5.2 Cardiovascular risk factors

CVRFs are linked to the appearance of vascular dementia and AD [120,121]. Epidemiological studies from the last decade suggest reductions in the prevalence of common CVRFs (smoking, high cholesterol, high blood pressure), while increasing prevalence of obesity and namely type 2 diabetes [122], pointing to the latter as the current major risk factor for cardiovascular diseases.

Patients with type 2 diabetes, have a greater risk of suffering from vascular cognitive impairment and dementia and late-onset AD in the elderly [123], [124], [125], but the underlying mechanisms are rather unknown. Novel DTI studies on type 2 diabetes have pointed to WM changes as a possible cause of cognitive impairments in these patients [126], and have confirmed the fornix as one of the impaired structures [26], [27], [28].

Moreover, either in obese (otherwise healthy) or non-obese elder people, body mass index negatively correlated with WM integrity, namely in the fornix and posterior regions of the corpus callosum [23], [24], [25]. The relationship between body mass and WM integrity could be related to altered vascular/inflammatory patterns, particularly in posterior regions of the corpus callosum and the fornix [113]. According to Bettcher et al., the greater impact of vascular and inflammatory factors associated to obesity on these two WM regions could be related to their marked metabolic demands and higher susceptibility to inflammatory processes in comparison with other WM fibers. Using MRI indices sensitive to demyelination and neuroinflammation (macromolecular proton fraction and k_f_) from qMT, Metzler-Baddeley et al., [127] found that in obese patients, abdominal visceral and subcutaneous fat area fractions were associated to microstructural changes in the fornix, but not in other regions studied such as parahippocampal cingulum, uncinate fasciculus, corticospinal tract, and the hippocampus. No differences in neurites dispersion were found with NODDI. Other studies, however, have found lower neurite density in the fornix associated with higher body mass index in cognitively normal obese patients [128].

### 5.3 Heart failure

Populations with heart failure have lower cardiac output, and the reduced blood flow pumped by the heart seems to have a detrimental impact on cerebral circulation [129], which may be associated to cognitive decline [130]. Interestingly, several studies have shown that cardiac dysfunction, in particular diastolic dysfunction, correlates with the severity of WM changes and cognitive decline [131], [132], [133]. Cardiovascular dysfunction has been related to reduced cerebral blood pressure leading to endothelial dysfunction and a decrease in nitric oxide (NO) production. Chronic depletion of NO biosynthesis induces oxidative stress and leads to pericytes loss, BBB disruption and neuronal toxicity [134]. On the other hand, oxidative stress induces pro-inflammatory mechanisms that exacerbate microvascular disease, and thus, aggravating WM damage [135,136].

Kumar et al., found that the volumes of the mammillary bodies and the fornix were significantly reduced in patients with heart failure manifesting spatial and working memory deficits [29]. Those differences remained after controlling for age, gender and intracranial volume. This would confirm the vulnerability of the fornix to cerebrovascular alterations.

### 5.4 Traumatic brain injury

The most relevant consequences of TBI are diffuse axonal injury caused by mechanical forces (shear stress) and cerebrovascular dysfunction (microhemorrhages, focal ischemia and edema) [137,138]. Namely, vascular pathology is associated with prolonged inflammation, BBB disruption, progressive WM damage and long-term neurodegeneration and disability [139,140]. Independently of the extent and location of the impact, TBI-related chronic inflammation is particularly evident in WM of subcortical regions and specially in the fornix [31], which could be explained by its high vulnerability to microvascular pathology and inflammation that follows TBI [139,141]. WM inflammation and degeneration persist for many years after trauma and likely increases the risk of developing AD later in life [142,143]. The key role of WM inflammation in cognitive impairments after TBI has been widely studied in patients.

Adult TBI patients (∼40 years old), showed persistent microglia activation ([11C](R)PK11195 (PK) binding in positron emission tomography imaging) in the internal capsule, putamen and, especially, in WM surrounding thalamic nuclei (fornix). However, only microglia activation in the latter correlated with lower executive function performance [144]. Another study in chronic TBI patients (∼26 years old) observed decreased FA in main WM tracts such as corpus callosum, internal and external capsule, superior and inferior longitudinal fascicles, and the fornix [30].

Intriguingly, in adolescent mild TBI patients (∼15 years old), Yallampalli et al., found an unexpected increased FA in acute phases, compared to controls, which they attributed to cytotoxicity and inflammation preceding myelin degeneration at more chronic phases [31]. This increased FA was associated with decreased processing speed in the Automated Neuropsychological Assessment Metrics Battery.

These studies strongly support that cognitive deficits in TBI individuals are related to network dysfunction and disconnection and could facilitate the appearance of AD later on [145,146].

## 6 Cerebrovascular disease and chronic white matter inflammation as a potential fornix modifier

Among the molecular and physiological mechanisms proposed as initiators of retrogenesis, cerebrovascular disease and neuroinflammation could be especially relevant considering the well-known susceptibility of WM to these conditions [83].

**Fig. 2:**
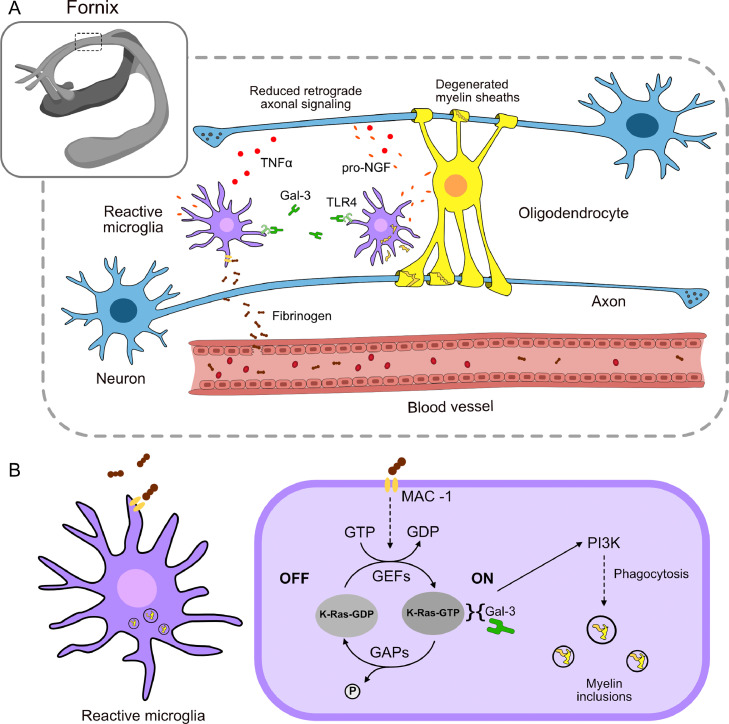
Schematic representation of a hypothetical model of fornix degeneration due to risk factors of Alzheimer's disease. A) Blood-brain barrier permeability induces inflammation of the white matter. The readjustment of endothelial tight junctions in response to a compromised blood microcirculation facilitates the extravasation of fibrinogen, capable of activating microglia. Reactive microglia can maintain and extend inflammation by releasing Gal-3 acting through TLR4. Reactive microglia phagocyte degenerated myeline and releases pro-NGF and TNFα that may impact oligodendrocytes viability and reduce retrograde axonal transport, thus, neuronal survival. B) Phagocytic phenotype in microglia is sustained by Gal-3 expression. Fibrinogen promotes microglia activation through binding the MAC-1 surface receptor. Gal-3 acts as a molecular switch that up-regulates and prolongs PI3K activity maintaining a phagocytic phenotype in microglia. Gal-3; galectin-3, TLR4; toll-like receptor 4, TNFα; tumor necrosis factor alpha; I3K; phosphatidylinositol 3-kinase; NGF, nerve growth factor; GTP, guanosine triphosphate; GDP, guanosine diphosphate; GEFs, guanine nucleotide exchange factors; GAPs, GTPase-activating proteins.

Cerebrovascular pathology and chronic WM inflammation has been observed in animal models of different conditions known to increase the risk of dementia: global cerebral ischemia (mimicking heart failure), aging, obesity, hypercholesterolemia, type 2 diabetes, and diffuse axonal damage after TBI [147], [148], [149], [150], [151], [152], [153], [154], [155]. Arterial stiffens and intermittent/chronic hypoxia lead to remodeling of the microvasculature and inflammation in WM [7,156]. Microvasculature remodeling induces readjustments in tight junctions of endothelial cells favoring blood-brain barrier (BBB) leakages and extravasation of blood components such as fibrinogen, a blood coagulation protein that promotes microglia activation through binding the CD11b-CD18 (MAC-1) surface receptor (Fig. 2A) [157], [158], [159]. Although neuroinflammation is intended as a protective process, chronic inflammation can be more harmful than beneficial [160].

Interestingly, all the above-mentioned animal models of risk factors of AD have reported the chronic activation of a subset of microglia expressing Galectin-3 (Gal-3, formally known as MAC-2) in the WM. Increased Gal-3 positive microglia has been associated with cognitive deficits in some models, for example, in aged rhesus monkey or hypercholesterolemic and diabetic mice [149,154,155]. Moreover, the presence of microgliosis in the WM, and namely Gal-3 positive microglia, is associated with a deficient electrical impulse transition in WM fibers [161,162], which suggests a detrimental impact on WM function. However, the causation still must be investigated.

Gal-3 is a β-galactoside-binding lectin that, presumably, promotes myelin-phagocytosis in MAC-1-activated microglia [163]. Namely, it acts as a molecular switch that up-regulates and prolongs K-Ras-GTP-dependent phosphatidylinositol 3-kinase (PI3K) activity (Fig. 2B) [163]. Gal-3 also targets the cytoskeleton, regulating microglia phenotype from branched (non-phagocytic) to amoeboid and phagocytic [164,165]. Moreover, as confirmed in several murine models of neuroinflammation (global brain ischemia or lipopolysaccharide (LPS)-induced inflammation), Gal-3 released by microglia seems to act as an endogenous paracrine toll-like receptor 4 ligand, prolonging the inflammatory response in the brain by sustaining microglia activation [150]. Accordingly, depletion of Gal-3 exerted neuroprotective and anti-inflammatory effects in these models.

On the other hand, some groups have proposed that Gal-3 microglia activation follows oligodendrocyte or myelin abnormalities, and play a role in myelin sheath reparation by releasing nerve growth factor (NGF) and phagocytosing the degenerated myelin [148,166]. NGF is a neurotrophin implicated in cell survival/proliferation and angiogenesis. It also plays an important role in axonal regeneration by promoting differentiation of oligodendrocytes and facilitating migration and proliferation of oligodendrocyte precursor cells to sites of myelin damage. However, others have reported that microglia are instead actively implicated in oligodendroglial dysfunction and death by releasing the precursor form of NGF (proNGF) upon LPS-induced WM inflammation [167,168]. The lack of specificity of some anti-NGF antibodies for labeling the immature or mature form of the neurotrophin has been an important confounding factor in the literature over the years [169]. Therefore, whether Gal-3 microglia express proNGF and not NGF, which will have a detrimental effect on WM structure, remains elusive.

NGF has a high affinity for TrkA/p75^NTR^ receptor, which mediates its trophic effects within the brain, while proNGF has a similar affinity for TrkA/p75^NTR^ receptor and sortilin/p75^NTR^ complex, the latter mediating the apoptotic pathway of the neurotrophin (e.g., oligodendrocyte death, acellular vessels, BBB disruption, neuronal death). Under pathological conditions, an imbalance in TrkA vs p75^NTR^ receptor ratios increases the sortilin/p75^NTR^ complex formation responsible for the proNGF switch from neurotrophic to apoptotic activity [170,171]. In this context, the overproduction of proNGF by reactive microglia would be fatal, especially within the fornix, which has shown an increased microglial activation capacity in response to stressors compared to other WM regions [172].

The p75^NTR^ receptor is highly expressed by neurons and glial cells under pathological conditions such as mechanical damage, focal ischemia, stroke, diabetes and AD, which may promote the apoptotic path of proNGF [173,174]. In fact, it has been shown that proNGF released by activated microglia in spinal cord injury mediates oligodendrocyte cell death by binding p75^NTR^ [167]. Hence, it would be interesting to understand whether the combination of an increased expression of proNGF by microglia and p75^NTR^ receptor in target glial cell populations could explain some aspects of WM degeneration in risk factors of AD and if these mechanisms are also valid in the context of AD.

In addition to myelin degeneration and oligodendrocyte death, proNGF and the pro-inflammatory cytokine tumor necrosis factor α (TNFα) released by microglia, are suspected to impair retrograde axonal transport causing neuronal dysfunction and death over time (Fig. 2A) [175].

## 7 Concluding remarks

Changes in lifestyle, early detection of CVRFs in midlife populations, and therapeutic interventions targeting the brain vasculature, therefore ensuring healthy hemodynamics, may constitute effective avenues for the clinical management of populations at high risk of suffering from late-onset AD with a vascular component.

Understanding what triggers WM changes, and the link with vascular pathology, will shed light on the relationship between the cerebrovascular dysfunction seen in many risk factors of AD and the neurodegeneration finally leading to cognitive decline. Namely, early detection of fornix degeneration in patients with CVRFs or TBI could help prevent the development of cognitive deficits or dementia later in life.

On the other side, understanding what causes WM pathology in the fornix, and the cascade of events leading to WM dysfunction, could help the optimization of techniques such as DBS-f for the treatment of cognitive decline, as well as other pharmacological or nonpharmacological approaches aiming to halt WM degeneration. For this reason, more research is necessary to reveal the specific cellular and molecular mechanisms causing WM damage in risk factors of AD.

## CRediT authorship contribution statement

**María Lacalle-Aurioles:** Writing – review & editing, Conceptualization. **Yasser Iturria-Medina:** Writing – review & editing.

## Declaration of Competing Interest

On behalf of all authors, the corresponding author states that there is no conflict of interest.
